# Did video kill the XR star? Digital trends in medical education before and after the COVID-19 outbreak from the perspective of students and lecturers from the faculty of medicine at the University of Ulm

**DOI:** 10.3205/zma001497

**Published:** 2021-09-15

**Authors:** Robert Speidel, Achim Schneider, Jasmin Körner, Claudia Grab-Kroll, Wolfgang Öchsner

**Affiliations:** 1 University of Ulm, Faculty of Medicine, Office of the Dean of Studies, Competence Center eEducation in Medicine, Ulm, Germany; 2 University of Ulm, Faculty of Medicine, Office of the Dean of Studies, Ulm, Germany; 3 University Hospital Ulm, Cardioanesthesiology, Ulm, Germany

**Keywords:** medical education, digital teaching, e-learning, future

## Abstract

**Aim: **Using a comparison of digital teaching in medicine before and after the COVID-19 outbreak, the aim of the study was to examine how ad hoc digitization has changed (1) the design of digital teaching, (2) the attitudes toward and the capabilities of digital teaching and learning and (3) the future importance of individual digital teaching elements.

**Methods: **Students and lecturers from the Medical Faculty of Ulm were asked to voluntarily participate in online surveys during the summer semesters of 2019 and 2020. The data was subsequently analyzed from a longitudinal and cross-sectional view descriptively as well as by using t-tests and Chi^2^-tests. In addition, using regression analyses, the results were controlled for associations with age, study progress, and media affinity.

**Results:** In the summer semester 2019, 163 students (6.1% response rate) and 56 lecturers (11.5%) participated in the surveys. In the following year, the participation increased to 285 students (10.4%) and 64 lecturers (12.8%). Video-based teaching elements such as videoconferencing and lecture recordings were increasingly used after the COVID-19 outbreak and considered more significant for future teaching. In contrast, virtual reality, augmented reality and 360°-videos, grouped under the term extended reality (XR), are descriptively becoming less important. Most lecturers would like to teach more digitally even after the pandemic but fear a decrease in learning effectiveness and contact with students, who tend to prefer asynchronous learning opportunities.

**Conclusion: **Video-based teaching elements proved to be a low-threshold and time-efficient solution during the lockdown and were also recommended for future use. The XR technology has been put on the back burner for the time being, but in view of the increased digital teaching motivation and capabilities, it can be assumed that lecturers will recognize and use the potential of XR as soon as they have the freedom to design innovative teaching again.

## Zusammenfassung

**Zielsetzung: **Mit einem Vergleich zwischen der digitalen Lehre in der Medizin vor und nach dem Ausbruch von Covid-19 soll untersucht werden, wie die ad-hoc Digitalisierung (1) die Gestaltung der digitalen Lehre, (2) die Einstellung und Befähigung zum digitalen Lehren und Lernen, und (3) die zukünftige Bedeutung einzelner digitaler Lehrelemente verändert hat.

**Methodik:** Die Studierenden und Dozierenden der Medizinischen Fakultät Ulm wurden in den Sommersemestern 2019 und 2020 gebeten, freiwillig an Online Umfragen teilzunehmen. Die Daten wurden anschließend im Längs- und Querschnitt deskriptiv sowie anhand von t- und Chi^2^-Tests analysiert. Zusätzlich wurden die Ergebnisse mit Regressionsanalysen auf Zusammenhänge mit dem Alter, dem Studienfortschritt und der Medienaffinität kontrolliert.

**Ergebnisse:** Im Sommersemester 2019 nahmen 163 Studierende (6.1% Rücklaufquote) und 56 Dozierende (11.5%) an den Umfragen teil. Im Jahr darauf stieg die Teilnahme auf 285 Studierende (10.4%) und 64 Dozierende (12.8%). Videobasierte Lehrelemente wie Videokonferenzen und Vorlesungsaufzeichnungen wurden nach dem Ausbruch von COVID-19 verstärkt eingesetzt und für die zukünftige Lehre bedeutsamer erachtet. Virtual Reality, Augmented Reality und 360°-Videos, die im Begriff Extended Reality (XR) zusammengefasst werden, verlieren dagegen deskriptiv an Bedeutung. Die meisten Dozierenden möchten auch nach der Pandemie digitaler unterrichten, fürchten jedoch Einbußen in der Lernwirksamkeit und im Kontakt zu den Studierenden, die eher asynchrone Lernangebote präferieren.

**Schlussfolgerung:** Videobasierte Lehrelemente haben sich während des Lockdowns als niederschwellige und zeiteffiziente Lösung bewährt und sich auch für einen zukünftigen Einsatz empfohlen. Die XR-Technologie ist augenblicklich in den Hintergrund gerückt, mit Blick auf die gestiegene Motivation und Befähigung für das digitale Lehren ist jedoch anzunehmen, dass die Dozierenden das Potenzial von XR erkennen und nutzen werden, sobald sie wieder Freiräume für die innovative Gestaltung der Lehre haben.

## 1. COVID-19 and digital teaching in medicine

In mid-March 2020, federal and state representatives, together with universities, decided to suspend face-to-face teaching as much as possible to contain the spread of the COVID-19 pathogen [1]. Medical schools were thus faced with the challenge of offering most of the upcoming summer semester online. Within weeks, entire courses had to be converted to online formats with digital learning materials. As a result of this unprecedented ad-hoc transition, the digital medical education component is likely to be higher after the pandemic [2], [3]. Whether this push for digitization means that established or novel technologies will be increasingly used depends primarily on how digital solutions have performed during the lockdown. To discern a possible trend reversal, a comparison must be made between teaching before and after the COVID-19 outbreak.

In medicine, digital teaching before COVID-19 was mainly characterized by the use of traditional digital media [4], [5], which, in most cases, played a minor role as an optional addition to the curriculum [6]. Learning management systems such as Moodle were used to communicate and share written (e.g., lecture notes and slides) and video-based data (e.g., lecture recordings and instructional videos). In contrast to other degree programs, electronic examination systems were also increasingly used in medicine for summative learning assessments (e.g., tablet-based OSCE). In contrast, new technologies such as extended reality (XR), which combines virtual reality (VR), augmented reality (AR) and immersive 360°-videos, were still in the pilot phase. However, XR was seen as having great potential, especially since it facilitates learning and collaboration in simulations independent of time and location. For example, just days before the lockdown, the Horizon Report [7] predicted that the hype around XR [8] would be followed by its increased use for teaching purposes [9], [10], [11].

How digital teaching is designed and how successful it is depends, in particular, on the digital competence of the lecturers [5]. Without referring to current empirical evidence, it is commonly assumed that the medical teaching staff has significant knowledge gaps and uncertainties in dealing with digital technologies [12], [13]. In contrast, students, who, as “digital natives”, have largely grown up with the technological advances, tend to be familiar with digital media but rarely use them actively on their own in their learning process. Most medical students use digital media for their studies only when they are a mandatory part of a course [5]. Prior to the pandemic, almost half of the medical students stated that their contact with digital technologies in the teaching context was mainly limited to traditional digital media and e-exams [4].

The COVID-19 pandemic changed the digital usage patterns of lecturers and students, which means that predictions such as those in the Horizon Report may have become less valid. Previous surveys of Corona-related online teaching at the international [14], [15] and national [16], [17] levels omit the XR technology, offer little medicine-specific insight, and are limited to the period after the SARS-CoV-2 virus outbreak. To determine the impact of COVID-19 on digital teaching in medicine, this study compares the 2019 (SS19) and 2020 (SS20) summer semesters. The longitudinal comparison is intended to answer three questions from the perspective of students and faculty:

1. How has the use of individual digital teaching elements changed?
2. How have the attitudes toward and the capabilities of digital teaching and learning changed?
3. How has the importance of individual digital teaching elements changed for the future?

## 2. Method

In order to investigate these questions, all students and lecturers from the study programs in human and dental medicine at the Medical Faculty of Ulm were asked twice via circular mail to voluntarily participate in online surveys before and after the outbreak of COVID-19 (see table 1 (Tab. 1)). The surveys in the SS20 repeated the questions from the previous year and added individual items regarding the effects of ad-hoc digitization (e.g., *“I gained technical know-how by switching to online teaching in the summer semester 2020.”*). The items used were developed after a relevant literature review on the basis of general survey development guidelines [18], [19] and were checked for validity by subject matter experts from medicine, media didactics, and statistics. Participants answered the self-generated items in either a binary form (“*yes”* or *“no”*), as a percentage or with the help of 5-point Likert-type scales (e.g., “*disagree”* to *“agree”*). Sample items being cited throughout the text refer to the latter response format. Because the surveys were conducted anonymously, an individual case assignment between measurement points is not possible. The normally distributed data was descriptively analyzed longitudinally (SS19 vs. SS20) and cross-sectionally (faculty vs. students) and by using t tests and Chi^2^ tests inferentially. In addition, using linear regression analyses, the SS20 results were controlled for influences of the variables age, semester, and media affinity.

Parallel to the research questions, the results are presented in three sections, each subdivided into lecturers and students. The section (1) “digital teaching design” contains information from the lecturers about the proportion of digital teaching, the teaching elements used, and the resource of time (e.g., *“I don't have enough time to deal with digital teaching.”*). The students rated the usefulness of the teaching elements that were utilized and provided information about their learning behavior (e.g., *“I obtained further learning content on my own.”*).

In the second section, the (2) “attitude toward and the capabilities of digital teaching” are reported. Lecturers indicated, among other things, the subjective added value and effort of digital teaching (e.g., *“Creating a digital learning unit is more time-consuming and difficult for me than preparing a face-to-face event.”*). Students compared the digital learning process with face-to-face teaching (e.g., *“It is more difficult for me to learn from digital learning units than learning in a face-to-face setting.”*). Students and lecturers assessed the gain in media technology know-how and the fit of individual learning objectives for digital teaching.

The third section looks at the (3) “future of digital teaching”. Lecturers and students assessed whether the proportion of digital teaching has increased in a sustainable manner and which teaching elements would play a significant role in future teaching (*“How important do you consider the use of the following teaching and learning applications for the future?”*).

## 3. Results

### 3.1. Digital teaching design

#### 3.1.1. Lecturers

**Figure 1:**
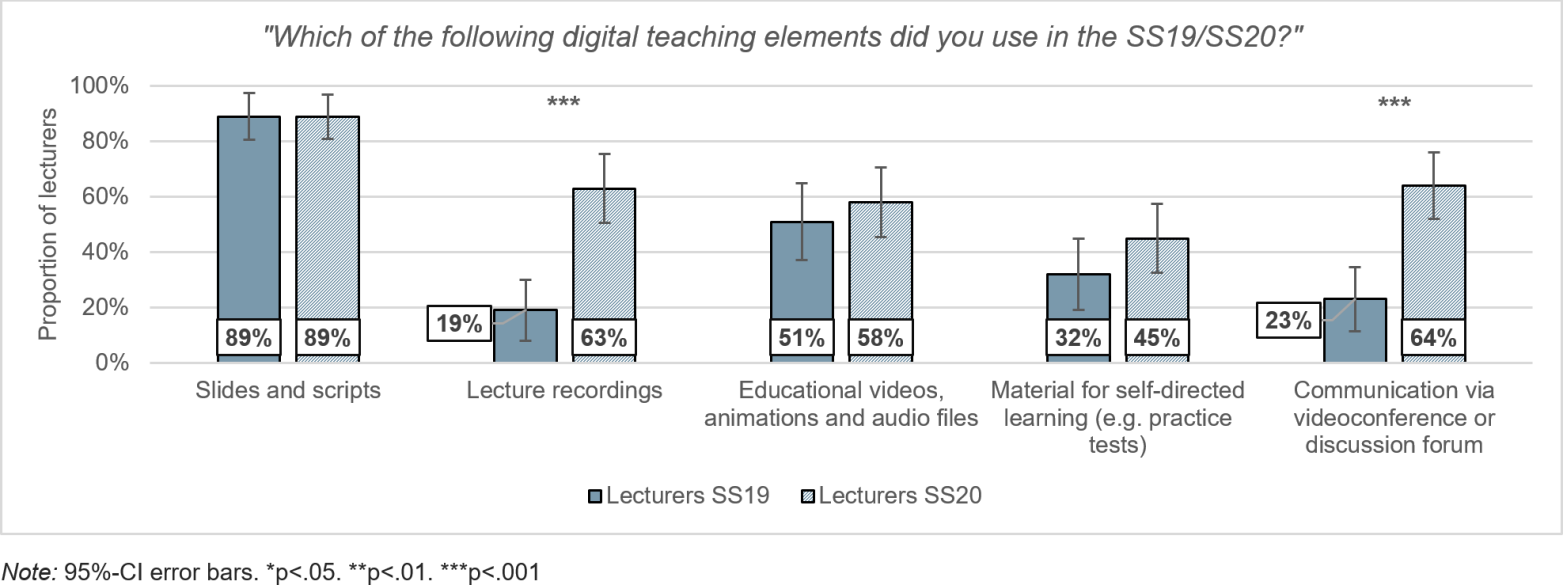
Percentage use of teaching elements

With regard to their total teaching load, 50% of the lecturers in the SS19 had assessed the digital portion of their teaching as (rather) low, while 21% had assessed it as (rather) high. Prior to COVID-19, this digital teaching component mainly consisted of supplementary teaching materials such as sets of slides and educational videos. Lecture recordings and web-based communication via video conference or discussion forums still played a subordinate role (see figure 1 (Fig. 1)).

In the online teaching during the SS20, lecturers were able to deliver an average of 67% (*SD*=23) of their teaching load digitally. In this ad-hoc digitization, lecturers in the SS20 (*M*=3.46, *SD*=1.23) had significantly more time for digital teaching (*t*(118)=2.47, *p*=.015, *d*=. 45) than in the previous year (*M*=2.89, *SD*=1.30). In addition to providing supplementary teaching materials, the time available was particularly invested in lecture recordings (*Χ^2^*(1)=21.18, *p*<.001, *φ*=.42) and communication via videoconference or discussion forums (*Χ^2^*(1)=17.87, *p*<.001, *φ*=.40), which were used significantly more often compared to the previous year (see figure 1 (Fig. 1)).

##### 3.1.2. Students

**Figure 2:**
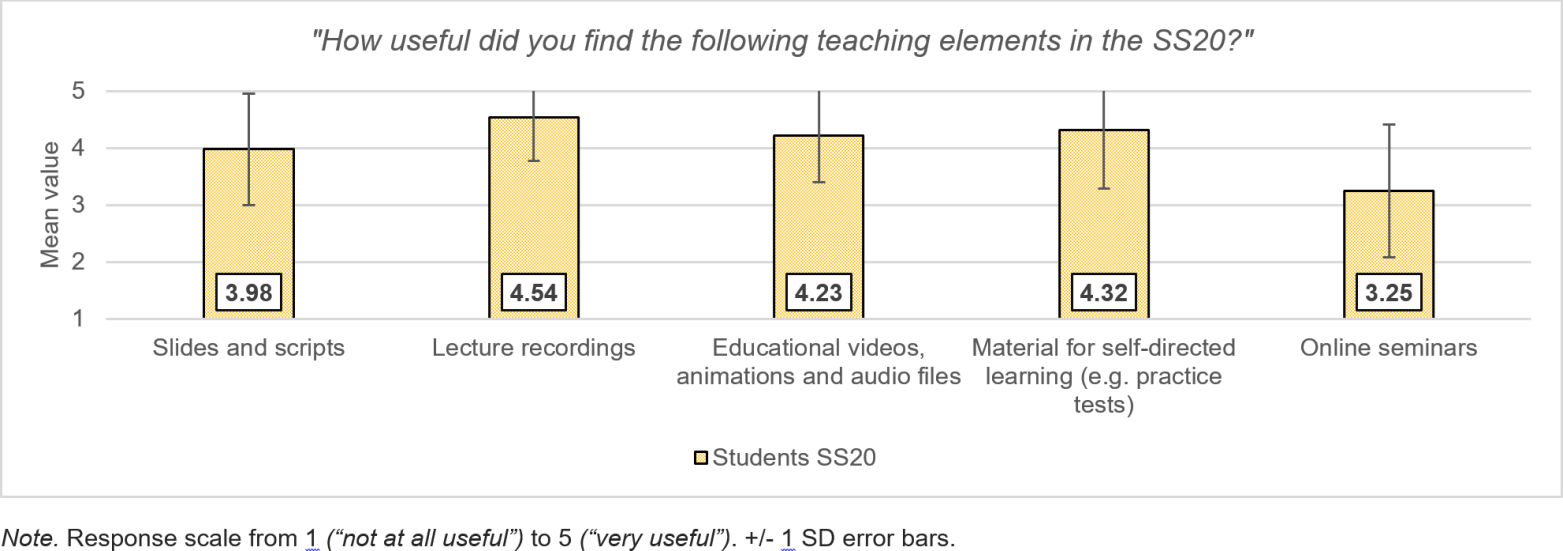
Subjective usefulness of teaching elements used

In the SS20, students rated the usefulness of the teaching elements used and indicated a preference for asynchronous teaching. Learning materials such as lecture recordings and slide sets that can be accessed regardless of time and location were descriptively considered more useful than online events on fixed dates (see figure 2 (Fig. 2)). The students stated they had worked on the self-study tasks provided (*M*=4.34, *SD*=. 93) and had partly supplemented them with external learning materials (*M*=3.69, *SD*=1.34). The medicine-specific learning platform “Amboss” was most frequently visited for external learning content (85%).

#### 3.2. Attitude toward and capabilities of digital teaching

##### 3.2.1. Lecturers

**Figure 3:**
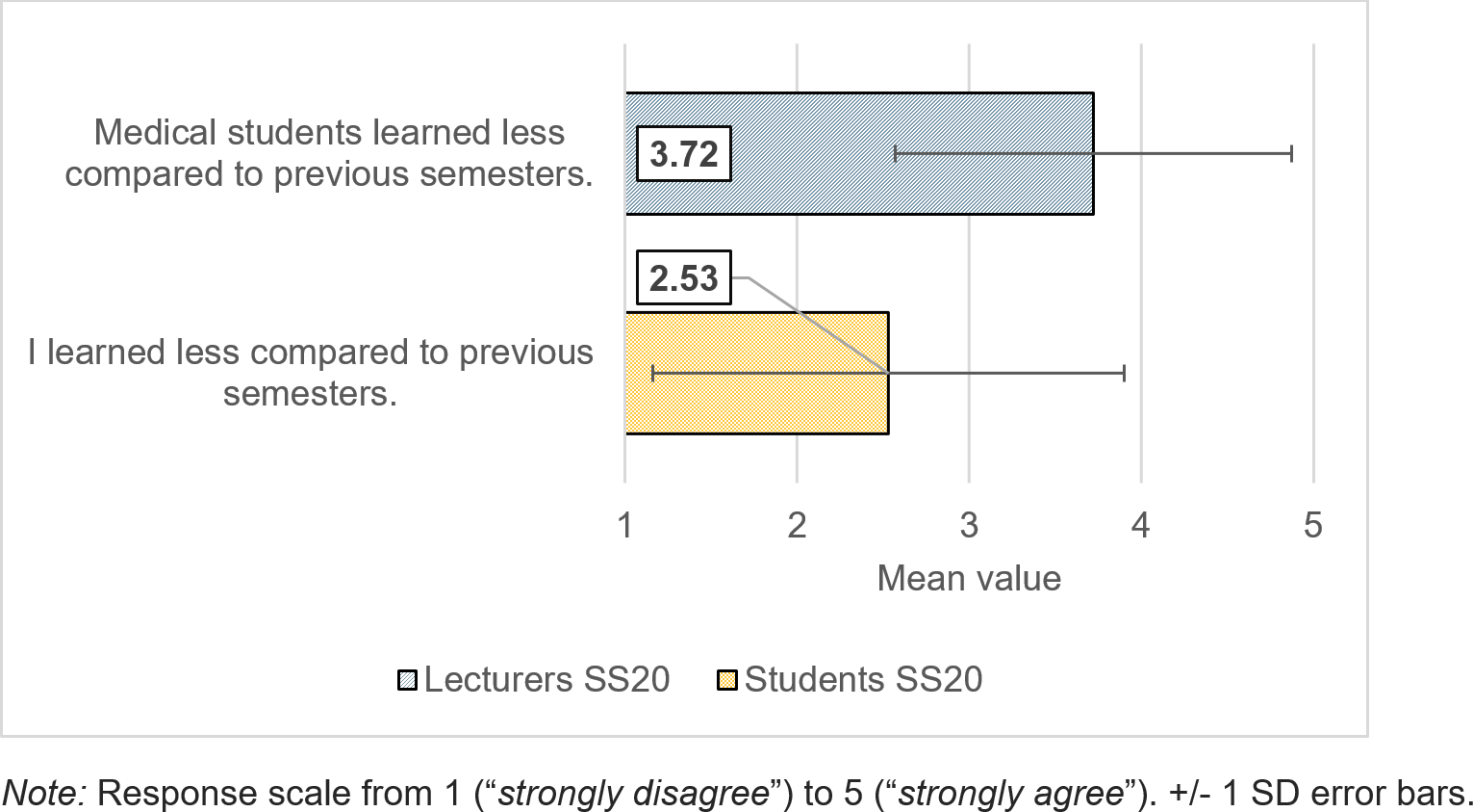
Subjective learning effectiveness

Many lecturers had considered digital teaching an enrichment in the SS19 (*M*=4.18, *SD*=. 94). This assessment did not change in the second survey (*M*=4.22, *SD*=.92). Nevertheless, 53% of lecturers said in the SS20 that they value digital teaching more than in the previous year, and 58% felt motivated to make their teaching more digital even after the pandemic. Nevertheless, 53% of the lecturers estimated the learning effect in the digital SS20 to be lower than in the regular teaching in the SS19 (see figure 3 (Fig. 3)). The fact that digital teaching was accompanied by limited contact with students was also noticed significantly more in the SS20 (*M*=4.50, *SD*=.84) (*t*(118)=7.45, *p*<.001, *d*=1.36) than in the SS19 (*M*=3.12, *SD*=1.12).

**Figure 4:**
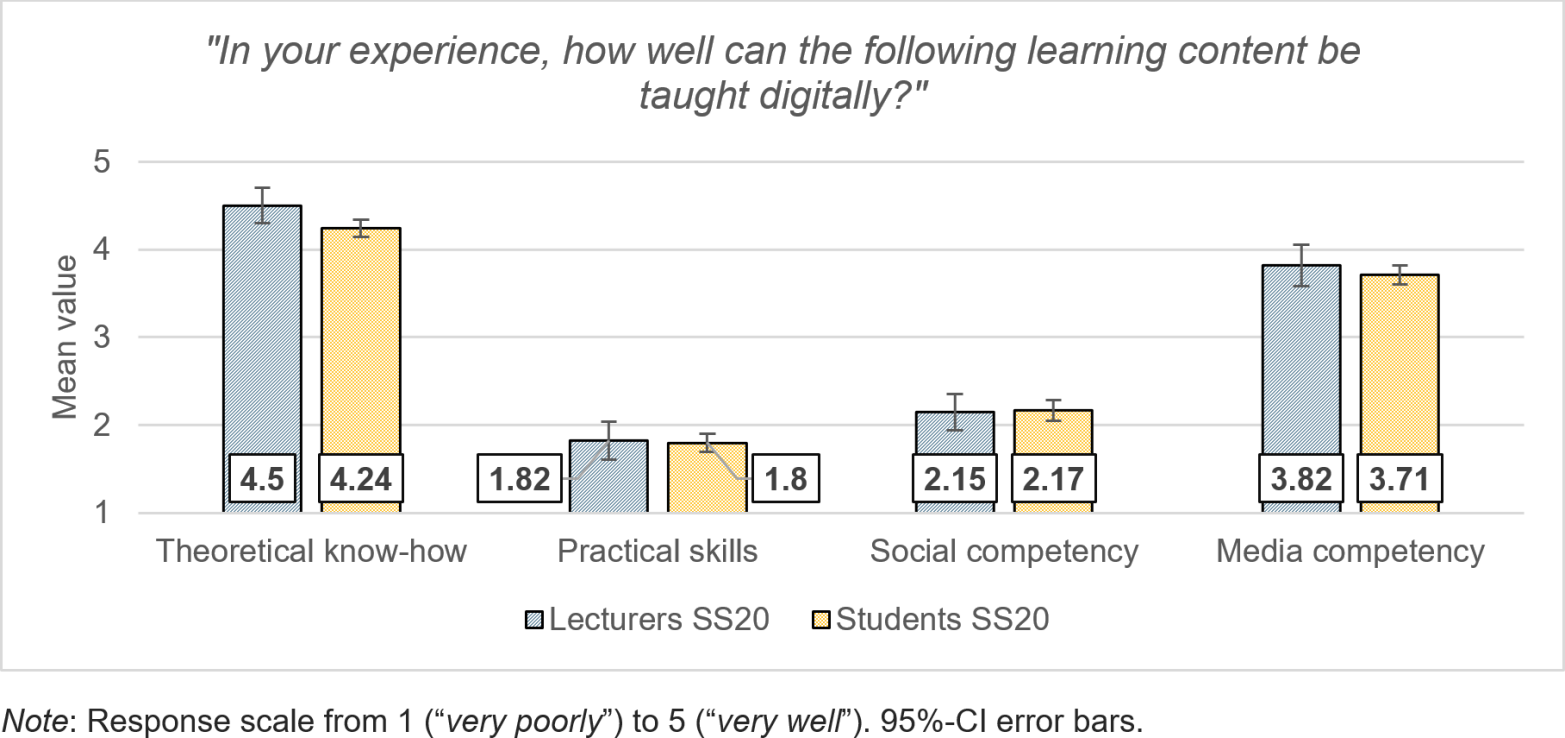
Suitable learning objectives for digital teaching

In the SS20, the lecturers saw the added value of digital teaching primarily in the transfer of theoretical knowledge and media competence. Practical skills and social competence, on the other hand, were deemed unsuitable as subject matters for digital teaching (see figure 4 (Fig. 4)).

As a result of the switch to online teaching, 77% of the lecturers also stated that they had gained in know-how in media technology. An indication of this gain is that the complexity of digital teaching was assessed significantly lower in the SS20 (*M*=2.11, *SD*=1.07) (*t*(118)=2.05, *p*=.042, *d*=. 38) than in in SS19 (*M*=2.54, *SD*=1.21). Nevertheless, the lecturers in the SS20 estimated the effort required for an online course to be higher than for a face-to-face course. This applied both to the creation (*M*=4.03, *SD*=1.12) and to the supervision (*M*=3.59, *SD*=1.24) of online courses.

##### 3.2.2. Students

Overall, students in the SS20 were undecided as to whether they found learning more difficult in a digital course unit than in a face-to-face course (*M*=2.92, *SD*=1.28), but only 28% felt that they had learned less in the digital SS20 than in the previous, predominantly analog semester (see figure 3 (Fig. 3)). For 46% of the students, the motivation for their studies was weaker than in the previous semester, and they estimated their gain in media technology know-how (*M*=2.59, *SD*=1.14) significantly lower as compared to the lecturers (*t*(347)=9.20, *p*<.001, *d*=1.27). In line with the lecturers, they saw the strength of digital teaching in the transfer of theoretical knowledge and media skills (see figure 4 (Fig. 4)).

#### 3.3. Future of digital teaching

##### 3.3.1. Lecturers

**Figure 5:**
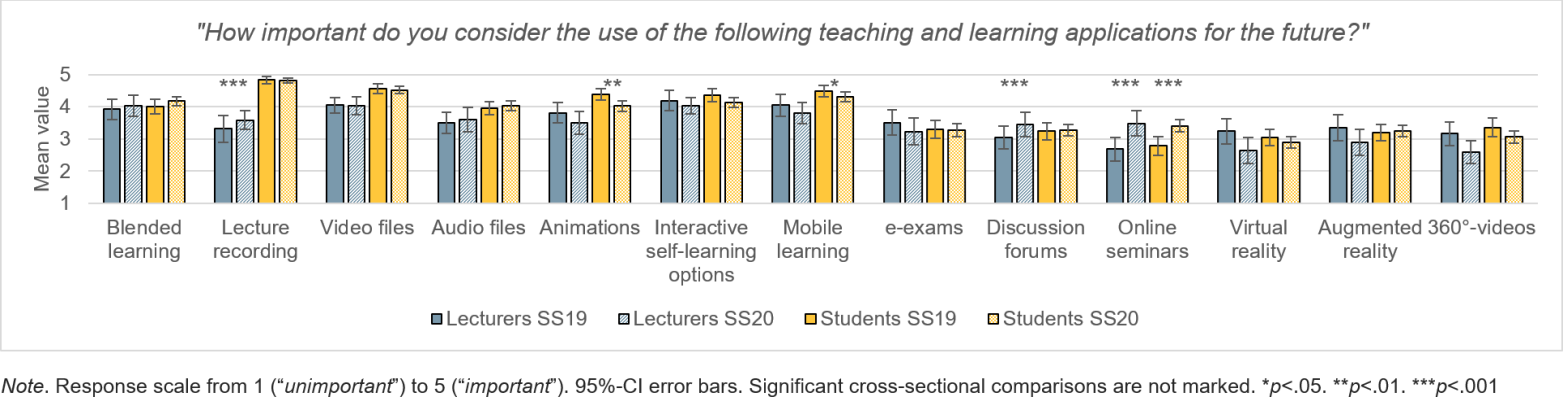
Future significance of individual teaching elements

With regard to future digital teaching, the lecturers in the SS19 considered supplementary teaching materials such as interactive self-learning options (e.g. online lessons), educational videos and mobile learning to be particularly important. Accordingly, they predicted a high relevance for the corresponding didactic concept of blended learning. The new XR technologies VR, AR and 360°-videos were predicted to make a moderate contribution to teaching. Online seminars seemed to be rather insignificant to them (see figure 5 (Fig. 5)).

During the restrictions of the Corona pandemic in the SS20, online seminars (*t*(115)=5.35, *p*<.001, *d*=.99), lecture recordings (*t*(118)=2.56, *p*<.012, *d*=.47) and discussion forums (*t*(112)=2.17, *p*=.032, *d*=.41) gained significantly in importance among lecturers. That the latter will replace traditional lectures in lecture halls was assumed by 28% of the lecturers. All other teaching elements – with the exception of audio files and the teaching concept of blended learning – descriptively lost significance (see figure 5 (Fig. 5)). This also applies, to a non-significant extent, to VR (*t*(102)=1.77, *p*=.079, *d*=.35), 360°-videos (*t*(94)=1.21, *p*<.229, *d*=.25) and AR (*t*(104)=1.06, *p*=.290, *d*=.21). Nevertheless, 84% of the lecturers estimated the future proportion of digital teaching in medical studies to be higher than before the pandemic.

##### 3.3.2. Students

Similar to the lecturers, the students before COVID-19 estimated that in the future supplementary teaching materials would play the most significant role in digital teaching designed as blended learning (see figure 5 (Fig. 5)). They did, however, attribute a higher importance to mobile learning (*t*(214)=3.42, *p*<.001, *d*=.54), video files (*t*(211)=3.90, *p*<.001, *d*=.61), audio files (*t*(208)=3.85, *p*<.001, *d*=.61), animations (*t*(207)=3.94,* p*<.001, *d*=.62) and especially lecture recordings (*t*(213)=11.58, *p*<.001, *d*=1.80) than the lecturers. They considered e-exams (*t*(202)=2.19, *p*=.03, *d*=1.21) to be less relevant, however. Like the lecturers, they attributed moderate importance to the XR elements for future teaching.

With regard to the experience during the online semester 2020, the future expectations of students differed (see figure 5 (Fig. 5)). Online seminars increased significantly in importance (*t*(414)=5.39, *p*<.001, *d*=.56) while animations (*t*(427)=2.78, *p*=.006, *d*=.28) and mobile learning (*t*(438)=2.36, *p*=.019, *d*=.23) decreased significantly in relevance. The immersive teaching formats of VR (*t*(389)=1.69, *p*=.091, *d*=.18) and 360°-videos (*t*(390)=1.75, *p*=.080, *d*=.18) also lost importance, although not to a significant degree. Students continued to consider lecture recordings most important with only 35% believing that they will replace traditional lectures. Compared to the lecturers in the SS20, they felt that animations (*t*(329)=2.74, *p*=.007, *d*=.40), mobile learning (*t*(339)=3.28, *p*<.001, *d*=.46), audio files (*t*(335)=3.59, *p*=.01, *d*=.50), video files (*t*(342)=3.77, *p*<.001, *d*=.53) and lecture recordings (*t*(345)=11.56, *p*<.001, *d*=1.6) were more important. Overall, 85% of students expected that the percentage of online teaching would be higher after the Corona crisis than before.

#### 3.4. Age, number of semesters and media affinity as control factors in the SS20

The statements made by the SS20 lecturers are partly related to their age (see table 2 (Tab. 2)). The older they were, the more likely they perceived the creation and supervision of a digital learning unit as additional effort compared to classroom teaching. The perception of digital teaching as an enrichment, on the other hand, decreases with age.

For SS20 students, age is positively related to the semester (*r*=.36, *p*<.001), but as a predictor, the semester is more appropriate contextually (see table 2 (Tab. 2)). The more advanced students were in their studies, the more likely they were to feel they had learned less in an online teaching format than in the previous semester, which still mostly consisted of face-to-face teaching. The blended learning concept is also rated as more important by students in higher semesters.

Media affinity helps to clarify interindividual variance among students and lecturers (see table 2 (Tab. 2)). For example, students with a high affinity for media had less difficulty learning digitally rather than face-to-face. Lecturers with a high affinity for media, on the other hand, found digital teaching less complicated.

## 4. Discussion

The outbreak of COVID-19 gave medical education an unprecedented digitization push. To determine how this ad hoc digitization in medicine has changed (1) the design of digital teaching, (2) attitudes toward and the capabilities of digital teaching and learning and (3) the future importance of individual digital teaching elements, we analyzed and compared the respective surveys completed by lecturers and students from the summer semesters of 2019 and 2020.

As expected, the proportion of digital teaching increased after the COVID-19 outbreak and, according to the lecturers and students interviewed, will not return to pre-pandemic levels. The increase was expressed primarily in the greater use of video-based teaching elements such as lecture recordings and online seminars, which played a much smaller role prior to the pandemic [4], [5]. This technically low-threshold solution approach was also pursued across courses at international universities [14]. Students generally accepted the resulting digital teaching that was offered but preferred asynchronously offered teaching content. The desire to determine when to learn and study seems to be particularly pronounced among medical students [20], which may be due to the high time demands of medical degree programs [21]. Due to the time pressure, medical students might prefer to learn superficially with videos and texts instead of anchoring their knowledge more deeply in time-intensive exchanges with fellow students and lecturers. Contrary to previous surveys [4], students in online studies not only passively consumed digital learning materials [5] but also independently accessed them on external platforms. Due to this independent design of the learning process, it appears that the students’ digital media use became more differentiated. 

The subjective value and appeal of digital teaching have also increased as a result of the lockdown, particularly among younger lecturers, which should strengthen the didactic use of digital media in the long term [5]. Across the generations, however, the limited contact with students was criticized, and a lower learning effectiveness was suspected. Lecturers may have found it difficult to assess, during digital exchanges on discussion forums or during video conferences, whether students understood the content. However, the students did not share the fear that they learned less digitally, even if their motivation for studying suffered as part of the social distancing during the SS20. Especially in the predominantly theoretical preclinical study section, digital teaching seems to be an adequate substitute; the omission of practical exercises, on the other hand, can hardly be compensated digitally.

According to lecturers and students, the future of digital teaching lies in the teaching concept of blended learning. This basic understanding did not change in the summer semester 2020 during which digital teaching was used not only as a support but also as a substitute. Especially students in the practically oriented clinical study section appreciated the synergetic combination of digital and analog teaching. By switching to online teaching, lecturers also increasingly found lecture recordings more valuable, while students had already considered them valuable prior to the pandemic. In line with the concept of blended learning, however, only a minority on both sides assumed that video recordings would replace traditional lectures.

The subjective relevance of XR technology, which was equally moderate among lecturers and students before COVID-19, descriptively decreased due to the pandemic, although XR could compensate for the deficits of digital teaching mentioned above. For example, practical and social skills that can hardly be taught with traditional media can be trained in collaborative VR simulations despite spatial distance [9]. Furthermore, XR conferences in which lecturers and students meet virtually as 3D avatars are an alternative to the communication in discussion forums and videoconferences, which lecturers find restrictive. However, in contradiction to pre-pandemic predictions [7], [8], [9], [10], [11], the potential of XR for future teaching was rated low. One explanation for this is that VR, AR, and 360°-videos have mostly been used only for individual pilot projects [5], so there is a lack of practical experience with the technologies to recognize and exploit their didactic potential. In line with this assumption, students consider individual teaching elements more important when they are available to them in their studies [22]. Moreover, the short-term transition to online teaching left little time for the introduction of creative approaches, so lecturers preferred to use familiar, video-based teaching elements. However, the relative disregard for XR is likely to be short-lived [23], [24]. The increased motivation for digital teaching rather suggests that the interest in XR will increase as soon as lecturers have time again to design their teaching not only pragmatically but also innovatively.

Since teaching resources are unevenly distributed at German universities [25] and the design of digital teaching depends on the attitudes and media competencies of individual faculty members [5], the time course of this development will depend on the respective location. In the same vein, the transferability of the results discussed must also be viewed critically since the surveys were conducted exclusively at the Medical Faculty of Ulm and participation was moderate. By providing the only comparison to date between digital teaching before and after the COVID-19 outbreak, the results, nonetheless, offer valuable insights into the short- and possibly long-term impact of the pandemic on medical education. Whether the observed trends will continue or reverse after the return of face-to-face teaching should be examined in future surveys.

## 5. Conclusion

During the lockdown, video-based teaching elements such as lecture recordings and online seminars proved to be a low-threshold and time-efficient solution for lecturers and students and thus recommend themselves for future use in teaching designed as blended learning. In pure online teaching, the lecturers lacked, above all, contact with the students, who preferred asynchronous teaching despite a loss of motivation. XR technology could be a solution, but at the moment it has been pushed into the background. However, in view of the increased motivation and capabilities for digital teaching, it can be assumed that lecturers will recognize and use the potential of XR as soon as they have time to develop innovative teaching designs again.

## Competing interests

The authors declare that they have no competing interests. 

## Figures and Tables

**Table 1:**
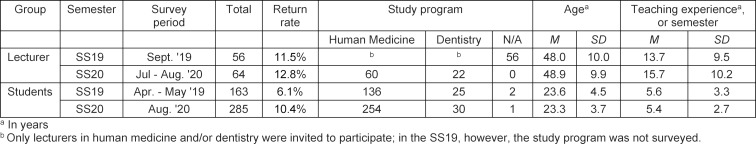
Sample description

**Table 2:**
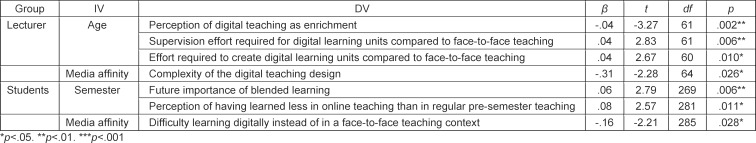
Linear regression analyses with the control variables age, semester, and media affinity
